# Characterization of the Stroke-Induced Changes in the Variability and Complexity of Handgrip Force

**DOI:** 10.3390/e20050377

**Published:** 2018-05-17

**Authors:** Pengzhi Zhu, Yuanyu Wu, Jingtao Liang, Yu Ye, Huihua Liu, Tiebin Yan, Rong Song

**Affiliations:** 1 School of Engineering, Sun Yat-sen University, Guangzhou 510275, China; 2 Guangdong Medical Devices Quality Surveillance and Test Institute, Guangzhou 510275, China; 3 Department of Rehabilitation Medicine, Sun Yat-sen Memorial Hospital, Sun Yat-sen University, Guangzhou 510275, China

**Keywords:** force modulation, stroke, grip control, fuzzy approximate entropy, root mean square jerk

## Abstract

*Introduction*: The variability and complexity of handgrip forces in various modulations were investigated to identify post-stroke changes in force modulation, and extend our understanding of stroke-induced deficits. *Methods*: Eleven post-stroke subjects and ten age-matched controls performed voluntary grip force control tasks (power-grip tasks) at three contraction levels, and stationary dynamometer holding tasks (stationary holding tasks). Variability and complexity were described with root mean square jerk (RMS-jerk) and fuzzy approximate entropy (fApEn), respectively. Force magnitude, Fugl-Meyer upper extremity assessment and Wolf motor function test were also evaluated. *Results*: Comparing the affected side with the controls, fApEn was significantly decreased and RMS-jerk increased across the three levels in power-grip tasks, and fApEn was significantly decreased in stationary holding tasks. There were significant strong correlations between RMS-jerk and clinical scales in power-grip tasks. *Discussion*: Abnormal neuromuscular control, altered mechanical properties, and atrophic motoneurons could be the main causes of the differences in complexity and variability in post-stroke subjects.

## 1. Introduction

The generation of human movement involves the activation and modulation of muscle force, and these functions are fundamental to performing daily activities. The force production capacity of muscles is an important indicator of motor function. In addition to force production capacity, the ability to modulate and sustain force at certain levels is also critical in daily use of the motor system. During the most-used upper extremity activity, reaching-to-grasp, for example, the ability to grip an object is the mark of the maturation of human motor behavior, requiring the force to be generated precisely at the safety margin predetermined by feedforward modulation [1,2].

Voluntary sensorimotor control function is deteriorated in most stroke survivors. After stroke, impairments such as spasticity [3,4], muscle weakness [5,6,7], increased reaction time [8], co-contraction [9], and contracture [10] lead to motor control dysfunction in patients [11,12]. The dysfunctions have been commonly found to impair force modulation during gripping. Strength is widely used as a measure of handgrip dysfunction. Boissy et al. [6] characterized upper extremity dysfunctions with maximal handgrip strength. Ada et al. [7] claimed that handgrip strength assessment was capable of directing interventions for improving muscle activity and avoiding spasticity. Sunderland et al. [5] also demonstrated that handgrip strength was a sensitive measure of stroke recovery within 6 months after stroke.

Although force production capacity is an important indicator for assessing stroke-induced impairment and recovery after training, force modulation is also critical for motor assessment, particularly for movements involving fine control. The variability of the generated force and kinematic parameters has been investigated in previous studies. Standard deviation and the coefficient of variation were applied to reflect the absolute and relative variability of force output in the study by Lodha et al. [13]. Some jerk-based indices were proposed to characterize the changed movement in subjects affected by chronic stroke [14], Huntington’s disease [15], or Parkinson’s disease [16], which were reported as less smoothness. Smoothness provides another aspect of variability: the intermittency in force maintenance induced by feedback control [17,18,19]. A dimensionless jerk index, the root mean square jerk (RMS-jerk), was provided to evaluate force smoothness [20,21].

Jerk-based parameters are linear indices of the variability of force output. Nonlinear measures, such as information entropy [22], approximate entropy [13,23,24], sample entropy [25,26], and fuzzy approximate entropy (fApEn) [27,28,29] provide a different perspective of force modulation than regularity. Hong et al. characterized the changed information entropy and ApEn of force output across different task settings to demonstrate modulated force during the procedure of motor adaptation [22,24]. In stroke survivors and aging people, the impaired motor system was also characterized by a smaller ApEn, which indicated more fixed force modulation, as demonstrated by Lodha et al. [13] and Vaillancourt and Newell [23]. The fApEn index, which was adapted from ApEn, was reported to be more robust and valid in complexity analysis, due to its fuzzy judgment of similarity [27].

In this study, voluntary grip force control at different levels (power-grip tasks) was evaluated, and stationary holding tasks were designed. The combination of power-grip tasks and stationary holding tasks may afford a comprehensive view of the force modulation most used in daily life. In the two tasks, the measures of variability and complexity, RMS-jerk and fApEn, respectively, as well as conventional indicators, i.e., force magnitude, Fugl-Meyer upper extremity assessment (FMA-UE), and Wolf motor function test (WMFT), were captured to characterize the impaired upper extremities of moderate and mild stroke survivors for two purposes: (1) to understand the stroke-induced changes under different kinds of force modulation in terms of variability and complexity of the grip force output, and the mechanisms underlying these changes in comparison to age-matched controls; and (2) to understand the relationship between clinical scales and the variability and complexity of grip forces.

## 2. Materials and Methods

### 2.1. Subject Selection

Eleven post-stroke subjects (mean age: 55.6 ± 16.1 years, 3 females, 8 males) and ten age-matched healthy subjects (5 males, 5 females, mean age: 51.7 ± 6.24) were recruited in this experiment. Basic clinical information related to the post-stroke subjects is shown in Table 1. The selection criteria of the stroke subjects included the following: (1) hemiparesis resulting from a single unilateral lesion of the brain with onset at least one month before data collection; (2) capability of voluntary grip contraction; and (3) intact cognition (mini mental state examination > 23). For the control group, all subjects were right-handed. All participants have written informed consent previously, and the study was approved by the ethical committee of Sun Yat-sen Memorial Hospital.

### 2.2. Apparatus and Procedure

**Figure 1:**
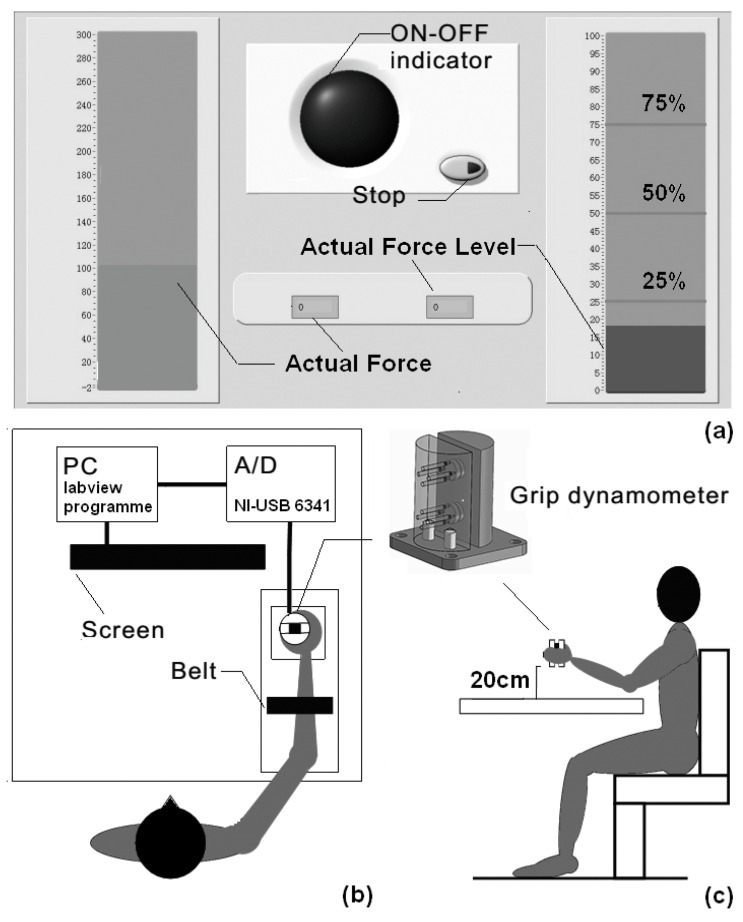
(**a**) LabVIEW interface; (**b**) Schematic diagram of the experimental setting and the execution of power-grip tasks; (**c**) Schematic diagram of the experimental setting and the execution of stationary holding tasks.

The customized-made grip dynamometer is displayed in Figure 1. Grip force was captured using force transducers (LSZ-F03B, Suzhou Battelle Automation Equipment Company, Suzhou, China) mounted inside the dynamometer. Data were saved to a personal computer using a 16-bit data-acquisition card (cDAQ-6251, National Instruments, Austin, TX, USA) at a sample frequency of 1000 Hz. Subjects were asked to sit and grasp the dynamometer on the table with their thumb and four fingers on opposite sides. During the experiment, their shoulder should be adducted at approximately 15°–20° of flexion, and their elbow at 90° of flexion.

As seen in Figure 1b, in order to prevent forearm motion, all subjects’ forearms were constrained with a belt during power-grip tasks. First, subjects were asked to generate a maximal grip force (MGF) for 5 s three times when the indicator was lit. From the three trials, the largest value of the MGF was obtained for the normalization of grip force during the next submaximal force level tasks. Then, each subject was asked to begin three different kinds of submaximal grip force control tasks (power-grip tasks; 25%, 50%, and 75% of MGF). A computer screen was provided to display real-time visual feedback (Figure 1a). On the screen, 25%, 50%, and 75% of the MGF were represented by three stationary horizontal red lines, and the actual force level was represented by a movable horizontal blue bar. The subjects should generate a suitable grip force to make the blue bar reach the red target line and persist for 5 s, during which the interface fed back the errors for grip force control. Each force level was performed three times, and to minimize fatigue, a 30 s rest was allowed for each subject after each trial. In stationary holding tasks, subjects were asked to grip the dynamometer at a height of 20 cm above the desk, and persist for 8 s. The stationary holding task was performed by each subject three times, and a 30 s break between each of the two trials was provided to avoid fatigue. All of the tests were conducted on the affected side of post-stroke subjects, and on the dominant side of the controls. All software used in the two tasks was programmed using LabVIEW (LabVIEW 2012, National Instruments, Austin, TX, USA). The upper-extremity motor impairments of the post-stroke subjects was assessed by clinical scales, namely, FMA-UE [30] and WMFT [31]. The modified Ashworth scale (MAS) was used to assess the information concerning the muscle tone in the upper extremities [32].

### 2.3. Data Analysis

The grip force was filtered with an 8th-order Butterworth low-pass filter (20 Hz). For power-grip tasks, the grip force was cropped into a 3 s window (1 s after timing started, and 1 s before termination) for further analysis, and for the stationary holding tasks, the force signal from the middle 6 s of the 8 s holding period was used.

The smoothness of the grip force was described with normalized RMS-jerk:

(1) $$\mathrm{RMS}-\mathrm{jerk}=\frac{\sqrt{\frac{1}{N}\sum {J(i)}^{2}}}{\mathrm{Mag}},$$

where *J*(*i*) is the jerk of the grip force at *i*-th sampling instant, which is the third derivative of the signal; and *N* is the total number of samples. Mag, the denominator, is a normalizing factor proposed by Hogan and Sternad [21], which is the mean maintenance force minus baseline.

The complexity of grip force was evaluated with fApEn. The computational process is summarized as follows.

Given a time series with *N* samples {*u*(*i*): 1 ≤ *i* ≤ *N*}, a vector sequence $X_{i}^{m}=\left\{u\left(i\right),\;L,\;u\left(i+m-1\right)\right\}-\frac{1}{m}\sum_{j=0}^{m-1}u\left(i+j\right)$ can thus be derived for the estimation of fApEn (*m*, *r*, *N*), which is the deviation of $\phi^{m}$ from $\phi^{m+1}$:

(2) $$\mathrm{fApEn}\;\left(m,\;r,\;N\right)=\phi^{m}\left(N,\;r\right)-\phi^{m+1}\left(N,\;r\right),$$

where the function $\phi^{m}$ indicates the averaged logarithm values across *j* of the averaged *m*-th similarity function across *i* for each *j* (between pairs of vectors $X_{i}^{m}$ and $X_{j}^{m}$ (*i* ≠ *j*)). Coefficients *m* and *r* determine the gradient and boundary of the membership function, and were empirically set to 2 and 0.15 [33,34], respectively. A discussion of coefficient optimization and a detailed description of fApEn computation have been provided in previous works [33,35]. All data analyses were accomplished using the Matlab signal process toolbox (Matlab R2014a, MathWorks Inc., Natick, MA, USA).

### 2.4. Statistics

The force magnitude, RMS-jerk, and fApEn outcomes of power-grip tasks were examined through two-way analysis of variance (ANOVA), with the assumption that stroke (affected side and age-matched controls) and the force level (25%, 50%, or 75% of MGF) were the two main factors. The results were further tested in a Bonferroni post hoc test as the effect of force level was significant. In addition, as the interaction effect was significant, the effect of stroke was tested with one-way ANOVA on the outcomes grouped at each force level. In stationary holding tasks, the force magnitude, RMS-jerk, and fApEn outcomes were tested with a two-tailed *t*-test to find the effect of stroke in observations. Additionally, Pearson correlation coefficients were applied to investigate the relationship between the clinical scales (WMFT and FMA-UE) and the two measures (RMS-jerk and fApEn) of force modulation, the course of which was followed by a significance test. The significance level of all statistical tests was set at 0.05, and SPSS 19 (SPSS Inc., Chicago, IL, USA) was used to accomplish all statistical computations.

## 3. Results

**Figure 2:**
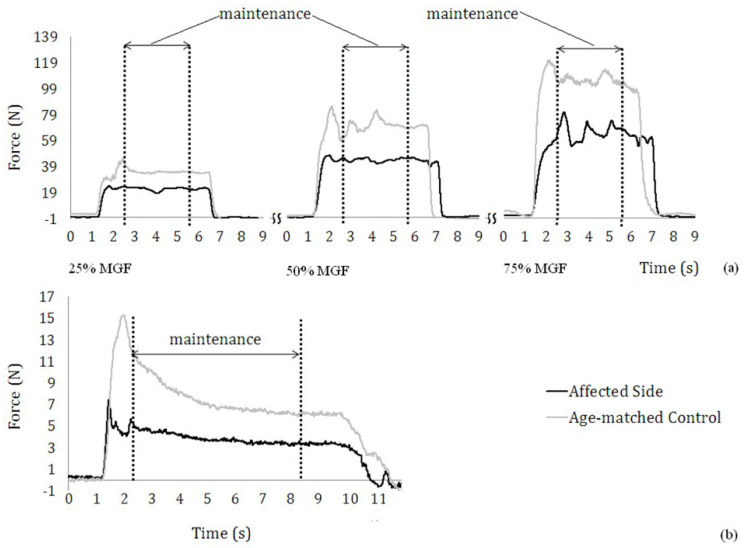
(**a**) Exemplar force profiles at 25%, 50%, and 75% maximal grip force (MGF) from power-grip tasks; the 3 s part in the middle of the whole 5 s process of force generation represents maintenance. (**b**) Exemplar force profiles from stationary holding tasks. The 6 s part following the initial 1 s during the whole 8 s process of force generation represents maintenance.

The MGF of post-stroke subjects and age-matched controls ranged from 17.68 to 142 N, and 121 to 246 N, respectively. An example of the force profiles from the two tasks for two subjects (a healthy subject and the affected side of a stroke subject) is shown in Figure 2. As shown in Figure 2a, the force outputs of age-matched controls were higher than those from the affected side of post-stroke subjects at all three force levels. Figure 2a,b display the force profiles from each group during power-grip tasks and stationary holding tasks, respectively. As seen in Figure 2a, the force outputs of age-matched controls were higher than those from the affected side of post-stroke subjects across three submaximal force levels. However, a similar trend was not found in stationary holding tasks, as shown in Figure 2b.

**Figure 3:**
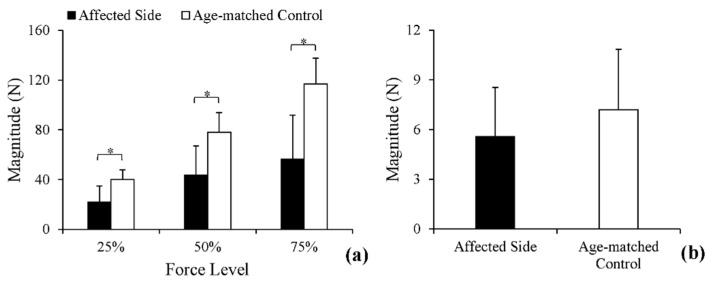
White columns: the magnitude of grip force of age-matched controls in the two tasks. Black columns: the magnitude of grip force of post-stroke subjects in the two tasks. (**a**) Comparisons of force magnitude at 25%, 50%, and 75% in power-grip tasks; (**b**) comparisons of force magnitude in stationary holding tasks. * Statistically significant difference (*p* < 0.05).

Mean force magnitude values in each group across the three submaximal force levels are illustrated in Figure 3a. In post-stroke subjects and age-matched controls, the force outputs increased monotonically as the force level increased. As was revealed from two-way ANOVA, the effect of force level was significant (*p <* 0.01) with the effect size (*ƞ^2^*) of 0.54. The following Bonferroni post hoc test demonstrated a significant difference between each of the two force levels (*p <* 0.05). The affected side of stroke subjects generated smaller forces at three submaximal force levels than age-matched controls. The effect of stroke was significant (*p <* 0.01, *ƞ^2^* = 0.52), however. The interaction effect was also significant (*p <* 0.01, *ƞ^2^* = 0.17). Further one-way ANOVA demonstrated that the effect of stroke was significant at all force levels (25%: *p <* 0.01, 50%: *p <* 0.01, 75%: *p <* 0.01). Additionally, the averaged force magnitude values from stationary holding tasks are demonstrated in Figure 3b. The force outputs of age-matched controls (7.19 N) were slightly higher than the affected side (5.57 N), but the effect of stroke was non-significant.

**Figure 4:**
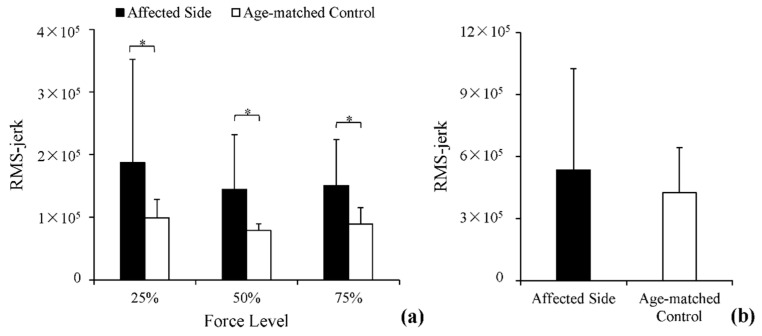
White columns: the RMS-jerk of grip force of age-matched controls in the two tasks. Black columns: the RMS-jerk of grip force of post-stroke subjects in the two tasks. (**a**) Comparisons of smoothness at 25%, 50% and 75% in power-grip tasks; (**b**) comparisons of smoothness in stationary holding tasks. * Statistically significant difference (*p* < 0.05).

RMS-jerk values of the force outputs from each group across the three submaximal force levels are displayed in Figure 4a. RMS-jerk values were greater in the affected side of post-stroke subjects than in age-matched controls. Two-way ANOVA identified a significant effect of stroke (*p <* 0.01, *ƞ^2^* = 0.16) and two other non-significant effects: force level and interaction effect. Similarly, in stationary holding tasks, RMS-jerk of the force output was slightly greater in the affected side of post-stroke subjects than in age-matched controls. However, the difference was non-significant, as demonstrated through a *t*-test (*p* > 0.05).

**Figure 5:**
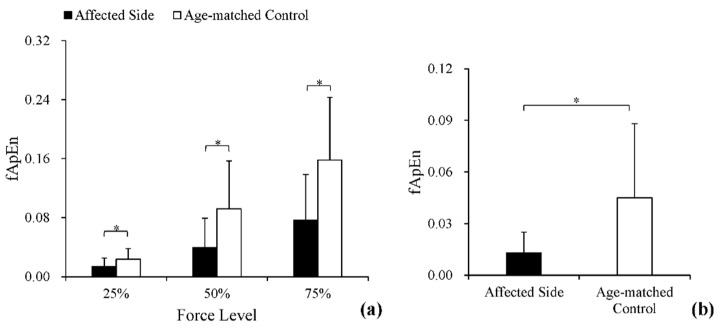
White columns: the fApEn of grip force of age-matched controls in the two tasks. Black columns: the fApEn of grip force of post-stroke subjects in the two tasks. (**a**) Comparisons of complexity at 25%, 50%, and 75% in power-grip tasks; (**b**) comparisons of complexity in stationary holding tasks. * Statistically significant difference (*p* < 0.05).

Figure 5a provides the fApEn values of the force outputs from each group across three submaximal force levels. The fApEn values increased monotonically as the force level increased, and the values of the affected side were smaller than those of age-matched controls. Two-way ANOVA revealed that the effects of force level (*p <* 0.01, *ƞ^2^* = 0.39) and stroke (*p <* 0.01, *ƞ^2^* = 0.19) were both significant, whereas the interaction effect was non-significant (*p* > 0.05, *ƞ^2^* = 0.08). Moreover, the following Bonferroni post hoc test also found a significant difference between each of the two force levels (*p <* 0.05). The fApEn values in stationary holding tasks are provided in Figure 5b. The values of the affected side were significantly smaller than those of age-matched controls (*p <* 0.05).

Table 2 shows the Pearson’s correlation coefficients relating the clinical scales with the RMS-jerk and fApEn values of the affected side. RMS-jerk was negatively correlated with FMA-UE and WMFT in terms of the force outputs at the three force levels in power-grip tasks (*p <* 0.05), whereas the correlation was weakened in stationary holding tasks. The correlations between fApEn and the two clinical scales were non-significant in the two tasks.

## 4. Discussion

In this study, grip control impairments were investigated by comparing post-stroke subjects and age-matched controls in terms of the variability and complexity of grip forces recorded in power grip and precision grip. The objective of this study was to find comprehensive measures of hand motor deficiencies in force modulation. As Ada et al. [7] suggested, maximal voluntary grip force reduction in the affected side was indicative of the weakness in force production that resulted from lost muscle cross-sectional area, and the reduction of motor units [5]. The significantly reduced force outputs at three force levels of the affected side indicated similar findings to those in previous studies.

### 4.1. The Variability of Force Modulation after Stroke

RMS-jerk reflects the variability of grip forces from the view of smoothness. The stroke-induced decrease in the smoothness of kinematic signals was previously reported in tasks with reflection [4,14]. These changes in smoothness may be visual feedback-dependent and could arise from the central nervous system. In studies of gripping [18], reaching [36,37] and multi-joint arm movement [38], greater neuromotor noise was reported in subjects after stroke. Neuromotor noise causes more errors in force output, and post-stroke subjects would rely more on feedback control to correct the errors [39]. As reported by Kim et al. [14], the decrease in smoothness was accompanied by an increase in the number of force corrections. The mechanical properties of the affected joints are other factors that lead to decreased smoothness. The deficient passive mechanical properties of joints were reported in post-stroke subjects while they were performing constant velocity stretch [40], sinusoidal excitation [41], and pendulum test [42]. Biomechanical model indices (stiffness, damping, viscoelasticity, and stretch reflex) were found to be significantly correlated to the Ashworth scale [40]. Changes in mechanical properties hinder precise force generation, and ultimately increase the variability in force output [42].

### 4.2. The Complexity of Force Modulation after Stroke

Entropy analysis of physiological signals has been widely applied as an indicator of frailty in aging [23,43], fatigue [44], stroke-induced hemiplegia [12,13], etc. A decrease in entropy values indicates a decrease in complexity or an increase in regularity. The reduced fApEn values in the affected side are in agreement with previous studies involving complexity analysis in chronic stroke [12,13]. According to Sethi et al.’s [12] study, the ApEn values of upper extremity kinematics were significantly reduced in post-stroke subjects during functional reach-to-grasp [12]. Lodha et al. [13] also observed stroke-related decreases in the ApEn values of isometric force in wrist–finger extension. Kang and Cauraugh [45] summarized these studies and suggested that stereotypic movements and underlying abnormal synergies were the causes that led to compromised motor adaptability across different task requirements [45,46]. Another mechanism attributed to the stroke-induced decrease in complexity is related to deficient central drive, the subsequent loss of alpha motor neurons, and, consequently, a decrease in the number and activation of the motor unit [34].

### 4.3. Clinical Implications

Although semi-quantitative clinical scales have been widely applied, ceiling effects have often been reported, especially for subjects with fine motor control changes [11]. The significant and strong correlations between RMS-jerk and clinical scales in power-grip tasks demonstrated the potential of RMS-jerk as a quantitative indicator of motor impairments. The significant stroke effect and non-significant force level effect in power-grip tasks also provided evidence of the stable difference between the two groups in RMS-jerk. Although there were non-significant correlations between the clinical scales and fApEn values, fApEn, as a nonlinear outcome measurement, describes the regular patterns of force modulation in the time domain, which reflect the expression of internal randomness rather than force amplitude [12,22]. The parameter could advance our understanding of motor impairments from a different aspect. Accordingly, the descriptions provided by RMS-jerk and fApEn involve two different perspectives of force modulation, which could reflect post-stroke impairments relatively comprehensively.

## 5. Conclusions

Two parameters, RMS-jerk and fApEn, were applied to analyze force modulation recorded in power and precision grip. These indicators described stroke-induced disabilities from the view of variability and complexity. Both indicators have the potential to be applied in a clinical setting for quantitative motor function evaluation.

## Figures and Tables

**Table 1:** Basic clinical information of post-stroke subjects.

Subject	Sex	Age (Year)	Duration (Month)	Affected Hemisphere	Assessment Scale		
					FMA-UE (0–66)	WMFT (0–75)	MAS (0–6)
1	F	63	3	R	61	62	1
2	M	40	4	R	63	67	1
3	M	22	1.5	R	51	56	1
4	F	52	2.5	L	64	71	0
5	M	73	1	L	48	50	0
6	M	64	2.5	L	60	57	0
7	M	49	2	R	65	73	0
8	M	72	6	R	65	72	0
9	F	63	5	R	42	46	0
10	M	59	4.5	L	56	60	1
11	M	37	2	L	46	48	3

Abbreviations: FMA-UE: Fugl-Meyer upper extremity assessment; WMFT: Wolf motor function test; MAS: modified Ashworth scale; F: female; M: male; R: right; L: left.

**Table 2:** Pearson correlation analysis.

		FMA-UE	WMFT
Power-Grip Tasks	fApEn		
	25% MGF	0.202	0.113
	50% MGF	0.211	0.290
	75% MGF	0.091	0.007
	RMS-jerk		
	25% MGF	−0.761 **	−0.689 *
	50% MGF	−0.869 **	−0.777 **
	75% MGF	−0.754 **	−0.712 *
Stationary Holding Tasks	fApEn	0.230	0.282
	RMS-jerk	−0.243	−0.250

Abbreviations: FMA-UE: Fugl-Meyer upper extremity assessment; WMFT: Wolf motor function test; fApEn: fuzzy approximate entropy; RMS-jerk: root mean square jerk. (* *p* < 0.05, ** *p* < 0.01).

## Abbreviations

The following abbreviations are used in this manuscript:

RMS-jerk	root mean square jerk
fApEn	fuzzy approximate entropy
FMA-UE	Fugl-Meyer upper extremity assessment
WMFT	Wolf motor function test
MGF	maximal grip force
ANOVA	analysis of variance
MAS	modified Ashworth scale
